# Beyond platinum sensitivity: immune-engaging therapies and multidimensional treatment stratification in relapsed small-cell lung cancer

**DOI:** 10.3389/fimmu.2026.1999309

**Published:** 2026-10-28

**Authors:** Yang Sun, Xutong Zhao, Jia Zhang, Qinghao Song, Miao Cheng, Ye Chen, Yuhan Liu, Wenyan Hou, Wanting Fu, Xu Zhang, Huanling Hao, Yi Zhao

**Affiliations:** 1 Department of Medical Oncology, The First Affiliated Hospital of Dalian Medical University, Dalian, China; 2 Department of Medical Oncology, Ningxiang People’s Hospital, Changsha, China; 3 The Clinical Skill Training Center of Dalian Medical University, Dalian, China; 4 Department of Medical Oncology, Dandong First Hospital, Dandong, China

**Keywords:** antibody–drug conjugate, lineage plasticity, platinum sensitivity, small-cell lung cancer, t-cell engager, treatment stratification

## Abstract

Although platinum sensitivity remains a convenient way to categorize relapsed small-cell lung cancer (SCLC), it no longer captures the full range of factors that may influence response to newer treatments. The interval to relapse still carries useful clinical information because it reflects the initial response, the pace of disease, and the emergence of resistance. It does not, however, identify whether a relapsed tumor can support checkpoint blockade, be efficiently recognized by a T-cell engager, or support delivery and respond to an antibody-drug conjugate (ADC). This Review considers treatment vulnerability through five interconnected features: lineage plasticity, antigen-presentation competence, T-cell function, surface-target availability, and susceptibility to the delivered payload. We integrate clinical and translational findings for DLL3-directed T-cell engagers, B7-H3-directed ADCs, and SEZ6-directed ADCs, emphasizing that resistance may arise from the target, the immune effector compartment, or the payload. We then introduce a Time-Immune-Target framework. Time represents relapse kinetics; Immune denotes the functional capacity to mount or redirect an antitumor immune response; and Target denotes the accessibility of a therapeutically relevant surface antigen. Prior therapy is superimposed on these dimensions as a record of evolutionary pressure. This proposal is intended to guide hypothesis development, not current treatment assignment. Its clinical value will need to be tested prospectively using serial tissue and blood sampling, imaging, and formal biomarker-by-treatment interaction analyses.

## 1 Introduction: from platinum sensitivity to the current biological state of relapsed disease

Rapid proliferation, early metastatic spread, and relapse after an initially treatment-responsive course define the clinical behavior of small-cell lung cancer (SCLC). For extensive-stage disease, platinum chemotherapy plus a PD-L1 inhibitor now forms a standard first-line backbone, but durable disease control remains uncommon. The central challenge after progression is therefore no longer a simple lack of available agents; it is how to choose and sequence therapies whose antitumor effects depend on different biological processes (1–3).

Chemotherapy-free interval (CTFI), platinum-free interval (PFI), and treatment-free interval (TFI) have long been used to distinguish platinum-sensitive, platinum-resistant, and refractory relapse. Thresholds such as 90 or 180 days are clinically convenient, but they were derived from different historical treatment settings and trial designs and do not imply a discrete biological transition at a specific time point. Reassessment of relapse cutoffs in the immunotherapy era further suggests that relapse timing behaves more like a continuous clinical variable than a stable molecular classifier (2–4).

This limitation has become more apparent with the emergence of therapies that depend on biological determinants other than platinum sensitivity. The randomized phase III DeLLphi-304 trial showed that the DLL3-directed T-cell engager tarlatamab improved overall survival after progression on platinum-based therapy, whereas the B7-H3-directed antibody–drug conjugate (ADC) ifinatamab deruxtecan (I-DXd) and the SEZ6-directed ADC ABBV-706 have shown antitumor activity in early-phase studies without standard-treatment control arms (5–7). The biological requirements for these modalities are not equivalent to continued platinum sensitivity. Platinum sensitivity therefore remains clinically useful, but it is unlikely to serve as a universal surrogate for treatment sensitivity across modalities.

SCLC is also biologically dynamic. Translational studies and analyses of relapsed samples suggest that transcriptional state, neuroendocrine differentiation, MHC class I (MHC-I) and antigen-presentation capacity, immune-cell infiltration, and surface targets such as DLL3, B7-H3, and SEZ6 may change under therapeutic pressure (8–10). Treatment history therefore records not only the sequence of prior therapies but also the selective pressures to which the tumor has been exposed.

These observations motivate a conceptual Time-Immune-Target framework (**Figure 1**). Time records the pattern of recurrence through PFI/CTFI, the depth of the earlier platinum response, and the subsequent pace of progression. Immune asks whether the recurrent disease can be seen and attacked by the immune system, incorporating antigen presentation, immune-cell access, and effector competence. Target asks whether the surface antigen required by a particular therapeutic platform remains available in the relapsed tumor. Prior treatment is layered across the framework because each exposure may shape later tumor states and resistance. The model is offered to organize evidence and define prospective research questions; it has not been validated for routine treatment assignment.

**Figure 1:**
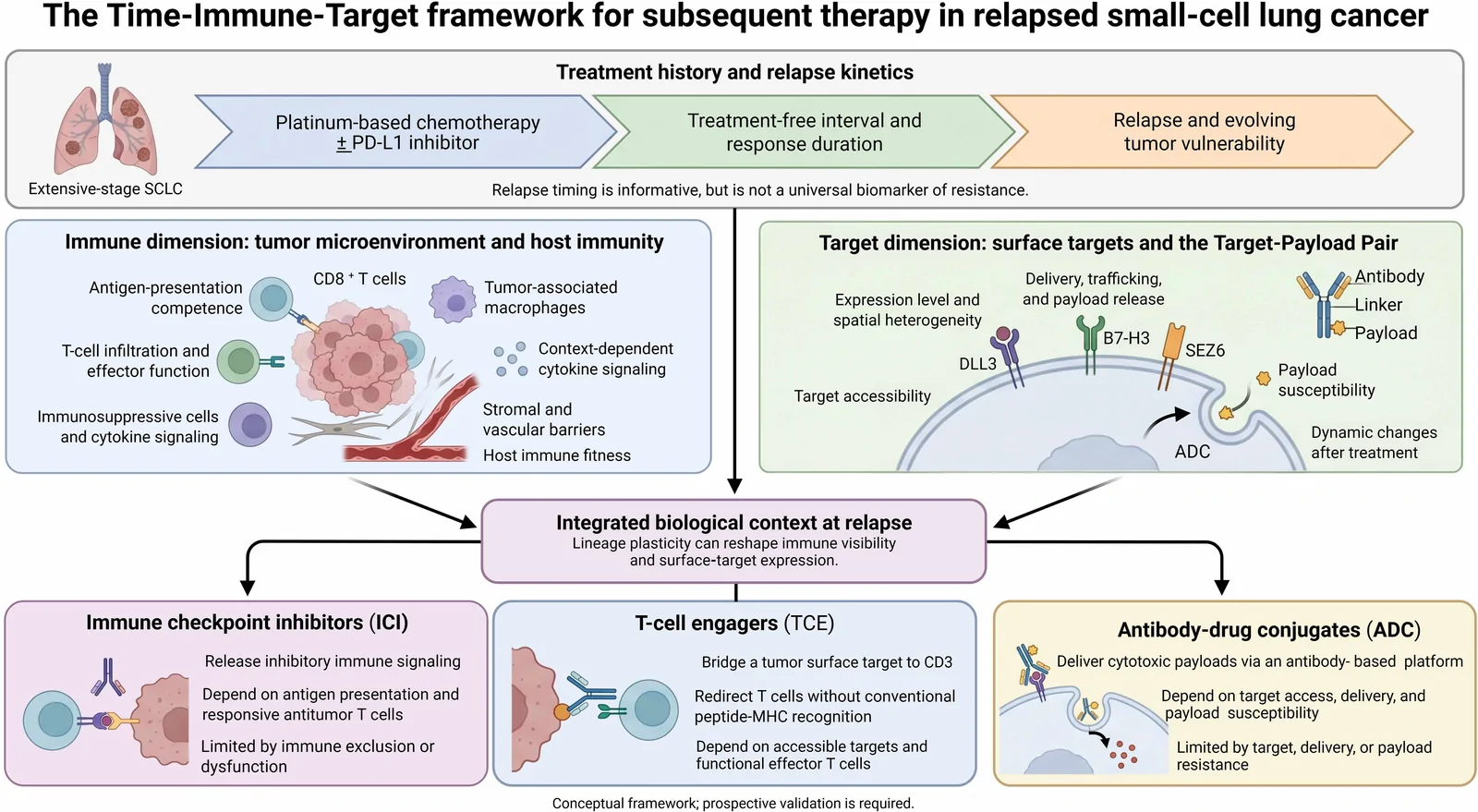
The time–immune–target framework for subsequent therapy in relapsed small-cell lung cancer. The time dimension integrates treatment history, response duration, and relapse kinetics; relapse timing is informative but is not a universal biomarker of resistance. The immune dimension encompasses antigen-presentation competence, T-cell infiltration and effector function, immunosuppressive cells and context-dependent cytokine signaling, stromal and vascular barriers, and host immune fitness. The target dimension incorporates surface-target expression and heterogeneity, accessibility, delivery and trafficking, payload release, and payload susceptibility within the Target–Payload Pair. DLL3, B7-H3, and SEZ6 illustrate relevant surface targets. Lineage plasticity can reshape both immune visibility and surface-target expression. These dimensions converge on the biological context at relapse and the distinct requirements of immune checkpoint inhibitors (ICIs), T-cell engagers (TCEs), and antibody–drug conjugates (ADCs). Internalization is illustrated as a common ADC delivery route, rather than a universal requirement for all platforms. The framework is conceptual and requires prospective validation. Created in BioRender. YANG, S. (2026) https://BioRender.com/jtbneek.

## 2 Methods: literature search and evidence selection

We conducted a narrative review designed to connect clinical evidence with treatment mechanism; the work was not structured as a systematic review or meta-analysis. PubMed searches were updated to 31 August 2026. The search strategy covered four linked areas: (i) SCLC at relapse, including recurrent disease, platinum-sensitive and platinum-resistant relapse, chemotherapy-free interval (CTFI), and platinum-free interval (PFI); (ii) immune phenotype, including MHC-I, antigen-presentation machinery (APM), SCLC-I, lineage plasticity, the immune microenvironment, and T-cell dysfunction; (iii) therapeutic modality and target, including DLL3, tarlatamab, T-cell engagers, B7-H3, ifinatamab deruxtecan, SEZ6, ABBV-011, ABBV-706, and ADCs; and (iv) acquired resistance and monitoring, including TOP1 payloads, circulating tumor DNA (ctDNA), circulating tumor cells (CTCs), molecular imaging, and biomarkers. We also screened the reference lists of pivotal trials, guidelines, translational reports, and recent reviews for additional studies.

Reports were included when they informed treatment mechanism, biomarker interpretation, resistance, or sequencing in relapsed SCLC. The greatest weight was given to clinical guidelines, randomized trials, prospective phase I/II studies, human translational datasets, and SCLC-specific mechanistic work. Reviews framed the field rather than supplying primary numerical efficacy estimates. Cross-tumor mechanistic evidence was included where SCLC evidence was limited and is explicitly identified as extrapolative. Preprints and unpublished data were excluded from the revised synthesis. Peer-reviewed case reports and small translational cohorts were retained only as exploratory observations, not as proof of efficacy or validated predictive biomarkers.

Evidence strength was matched to each claim. Comparative efficacy statements were based on randomized treatment comparisons where available; studies without standard-treatment controls described activity and toxicity; translational studies supported associations; and experimental work supported biological plausibility. Dose-randomized studies were distinguished from comparative treatment trials. Prognostic associations were separated from evidence of treatment prediction, and baseline candidates were distinguished from dynamic measurements and prior-exposure variables. During revision, targeted verification and reference-list supplementation addressed platinum mechanisms, chemoimmunotherapy, the TME, and DLL3 ADCs; this was not a new systematic search. The core literature cutoff remained 31 August 2026. A separately identified regulatory update of 25 September 2026 is included for I-DXd and is not used as efficacy evidence.

## 3 Platinum sensitivity: from historical clinical stratification to a starting point for modern treatment decisions

### 3.1 Why time-based stratification has remained clinically useful

Relapse interval has remained clinically useful in second-line SCLC because it integrates several features of prior disease behavior, including response to initial therapy, the tempo of resistance evolution, and overall disease aggressiveness. Time is therefore not a specific molecular biomarker; it is better viewed as a composite clinical surrogate.

In platinum-sensitive relapse, the randomized phase III trial by Baize et al. compared carboplatin plus etoposide with topotecan and reported median progression-free survival (PFS) of 4.7 versus 2.7 months, respectively (hazard ratio, 0.57), with objective response rates (ORRs) of approximately 49% versus 25%, supporting the continued clinical relevance of platinum rechallenge in patients with a longer relapse interval (11). In the single-arm phase II lurbinectedin study, activity was greater with longer CTFI: ORR was approximately 45% in patients with CTFI ≥90 days versus 22.2% in those with CTFI <90 days (12). In a prespecified subgroup with CTFI ≥180 days, ORR was 60.0%, disease control rate was 95.0%, and median duration of response (DOR), PFS, and overall survival (OS) were 5.5, 4.6, and 16.2 months, respectively; because this subgroup included only 20 patients, the findings describe activity in a long-CTFI population rather than provide predictive validation (13). The single-arm phase II study of liposomal irinotecan LY01610 reported an ORR of 32%, median PFS of 4.0 months, and median OS of 9.7 months among 66 patients with relapsed SCLC, 68% of whom had CTFI <90 days. These findings show that non-platinum cytotoxic activity can still be observed in a population enriched for early relapse, but they do not establish mechanism-specific sensitivity in this group (14). By contrast, the randomized phase III ATLANTIS trial did not show an OS improvement with lurbinectedin plus doxorubicin over topotecan or CAV, reinforcing that relapse timing cannot substitute for randomized evidence for a specific regimen (15). Selected clinical trials informing first-line treatment context and therapeutic decisions in relapsed SCLC are summarized in **Table 1**.

**Table 1:** Selected first-line contextual and relapsed SCLC trials.

Study	Treatment	Population/design	ORR	PFS	OS	Implication for stratification
IMpower133 (23)	Atezolizumab + carboplatin–etoposide vs placebo + chemotherapy	FIRST-LINE CONTEXT; randomized phase III; n=403	Not summarized	5.2 vs 4.3 mo	12.3 vs 10.3 mo	Supports initial chemoimmunotherapy; does not validate ICI rechallenge
CASPIAN (24)	Durvalumab + platinum–etoposide vs chemotherapy	FIRST-LINE CONTEXT; original phase III comparison; n=537	Not summarized	Not summarized	13.0 vs 10.3 mo	Initial OS benefit; not a relapsed-treatment comparison
Baize 2020 (11)	Carboplatin + etoposide vs topotecan	Platinum-sensitive relapse; randomized phase III	49% vs 25%	4.7 vs 2.7 mo	—	Time retains direct clinical value for platinum rechallenge
Trigo 2020 (12)	Lurbinectedin	Phase II; n=105	35.2% overall	—	—	Longer CTFI was associated with higher ORR in a single-arm study; not predictive validation
Subbiah 2020 (13)	Lurbinectedin	Prespecified CTFI ≥180 d subgroup; n=20	60.0%	4.6 mo	16.2 mo	Small prespecified subgroup supports an association between long CTFI and cytotoxic activity; interpret cautiously
CheckMate 331 (41)	Nivolumab vs chemotherapy	Randomized phase III; n=569	13.7% vs 16.5%	1.4 vs 3.8 mo	7.5 vs 8.4 mo	ICI did not improve OS in an unselected population
DeLLphi-301 (48, 49)	Tarlatamab	Phase II/regulatory analysis	≈40%	≈4.9 mo	—	Single-arm TCE activity is not explained by platinum sensitivity alone
DeLLphi-304 (5)	Tarlatamab vs physician choice	Randomized phase III; n=509	35% vs 20%	4.2 vs 3.2 mo	13.6 vs 8.3 mo	Randomized OS benefit established for DLL3 TCE
IDeate-Lung01 (6)	I-DXd 12 mg/kg	Phase II; randomized dose optimization then expansion; pooled 12-mg/kg n=137; no standard-treatment control	48.2%	4.9 mo	10.3 mo	B7-H3 ADC activity; no validated IHC threshold or comparative treatment effect
ABBV-706 (7)	SEZ6 ADC	Phase I with randomized dose optimization; SCLC monotherapy n=124; no standard-treatment control	52%	5.4 mo	11.3 mo	SEZ6 ADC activity; prior TOP1-inhibitor analysis was *post hoc*

Patient populations, treatment lines, follow-up, efficacy assessments, and statistical designs differ across studies. The data are presented to illustrate modality-specific evidence and stratification concepts and should not be used for unadjusted cross-trial ranking.

### 3.2 The 90-day and 180-day thresholds are not biological breakpoints

Definitions of sensitive relapse differ across guidelines and trials, leaving patients who relapse between 90 and 179 days in a familiar gray zone (1–4). Torasawa et al. re-evaluated PFI cutoffs in an immunotherapy-era cohort and found that the statistically optimal threshold may differ from conventional 60- or 90-day definitions (4). Rather than supporting replacement of one rigid cutoff with another, these findings underscore the continuous nature of relapse kinetics.

In historical chemotherapy studies, relapse timing and subsequent cytotoxic sensitivity often move in the same direction. This association, however, cannot be assumed to apply equally to therapies with different mechanisms. Clinical relevance is not the same as mechanistic specificity. Early relapse indicates a more aggressive clinical course and faster resistance evolution, but it does not establish universal resistance to T-cell engagement, ADC delivery, or other targeted mechanisms.

### 3.3 Repositioning rather than discarding time

Accordingly, an early relapse after platinum should not be extended into a blanket assumption that every later modality will fail. The relapse interval remains valuable for judging the likelihood of benefit from platinum rechallenge, estimating clinical risk, and stratifying trials. Its role in modern decision making is therefore foundational but circumscribed: it describes how the disease returned, not every vulnerability present in the tumor at the time of recurrence.

### 3.4 Molecular mechanisms of platinum action and resistance

Cisplatin and carboplatin generate reactive platinum species that bind DNA, predominantly producing intrastrand adducts, together with interstrand cross-links and other lesions. These lesions distort DNA and impede replication and transcription. Structural studies established the pronounced deformation induced by the major cisplatin–DNA adduct (16). Cytotoxicity consequently depends on intracellular drug exposure, the persistence of DNA damage, and the ability of a damaged cell to execute death programs. Clinical resistance after platinum–etoposide reflects selection by the combination and cannot automatically be assigned to platinum alone.

Reduced uptake, increased sequestration or export, and thiol-mediated detoxification can reduce the amount of platinum reaching DNA. Experimental work outside SCLC implicates the copper transporter CTR1 in uptake and ATP7B-dependent trafficking in platinum tolerance (17, 18). These are mechanistic examples rather than validated SCLC selection biomarkers. Direct SCLC cell-line experiments showed that increased glutathione availability was associated with cisplatin resistance and that glutathione depletion altered DNA platination and adduct repair (19). The relative contribution of each pathway in an individual relapsed tumor remains uncertain.

Resistance can also arise through more effective lesion repair or damage tolerance, failure of apoptosis, and epigenetic adaptation. In paired SCLC patient-derived models exposed to cisplatin–etoposide, Gardner et al. linked acquired resistance to EZH2-associated silencing of SLFN11 in a subset; EZH2 inhibition restored SLFN11 expression and enhanced chemotherapy activity (20). This supports a role for replication-stress responses without making SLFN11 a validated platinum-specific biomarker. More recent SCLC experiments associated USP22 loss with attenuated DNA-damage signaling and apoptosis and with chemotherapy resistance (21). Lineage plasticity and selection of drug-tolerant states can coexist with these mechanisms (10). Extrinsic stromal and immune influences are considered together in Section 4.3 to avoid conflating tumor-intrinsic resistance with the surrounding microenvironment.

### 3.5 Why platinum resistance does not imply universal therapeutic resistance

Platinum resistance is a clinical phenotype that can arise through several biological routes. Reduced platinum accumulation need not eliminate DLL3 or prevent CD3-mediated T-cell redirection. Conversely, retained platinum sensitivity does not demonstrate adequate antigen presentation for checkpoint blockade. TCEs depend on accessible target and competent effectors, whereas ADCs depend on target-mediated delivery and susceptibility to their particular payload. Shared DNA-damage tolerance could create overlap between cytotoxic resistance mechanisms, but platinum, calicheamicin, and TOP1 inhibitors do not produce identical lesions. Thus, neither universal cross-resistance nor complete independence should be assumed (5–7, 20, 22). These distinctions motivate mechanism-specific reassessment rather than abandonment of relapse timing.

### 3.6 Platinum-based chemoimmunotherapy and the immune dimension

Two pivotal randomized trials established the value of adding PD-L1 blockade to first-line platinum–etoposide in extensive-stage SCLC. In IMpower133, 403 untreated patients received atezolizumab or placebo with carboplatin–etoposide, followed by maintenance atezolizumab or placebo. At the primary analysis, median OS was 12.3 versus 10.3 months (hazard ratio, 0.70; 95% CI, 0.54–0.91), and median PFS was 5.2 versus 4.3 months (23). In the initial CASPIAN comparison, 268 patients received durvalumab plus cisplatin- or carboplatin–etoposide and 269 received platinum–etoposide alone; median OS was 13.0 versus 10.3 months (hazard ratio, 0.73; 95% CI, 0.59–0.91) (24). These estimates refer to the original analyses, not subsequent updates. The trials support the combination in untreated disease; they do not establish ICI rechallenge efficacy after progression or identify platinum sensitivity as an ICI-predictive biomarker.

Chemotherapy may release tumor antigens and danger signals, affect antigen-presenting cells, and change the conditions for T-cell priming and infiltration. These effects are context dependent. In colorectal experimental models, oxaliplatin elicited calreticulin-associated immunogenic death more effectively than cisplatin, demonstrating that immunogenicity cannot be generalized across platinum compounds or tumor types (25). The survival benefit of chemoimmunotherapy therefore does not prove that platinum consistently converts SCLC into an immune-inflamed tumor. Antigen release can coexist with deficient MHC-I presentation, immune suppression, or loss of effector cells. The Immune dimension captures the resulting functional state at relapse; the cellular and stromal processes that may shape it are detailed in Section 4.3 (8, 9, 26).

## 4 The immune ecology of SCLC: from molecular subtype and lineage plasticity to functional immune state

### 4.1 Molecular subtypes provide state coordinates rather than fixed labels

Based on ASCL1, NEUROD1, POU2F3, and inflammation-related transcriptional programs, SCLC can be described by major states including SCLC-A, SCLC-N, SCLC-P, and SCLC-I. Transcriptomic analysis by Gay et al. showed that SCLC-I is characterized by greater expression of immune-related programs and displayed an exploratory signal of greater immunotherapy benefit in IMpower133, but this subtype has not been prospectively validated as a treatment-selection biomarker (8). An analysis of long-term survivors from IMpower133 likewise identified enrichment of immune-related features and SCLC-I among long-term survivors, but these findings remain exploratory associations (27).

SCLC subtype is also plastic. Translational and model-based work by Mahadevan et al. found that an MHC-I-high state was more common in non-neuroendocrine phenotypes and was accompanied by greater immunogenicity and STING-related signaling; experimental models further suggested that therapeutic pressure can be associated with cell-state transitions (9). Analyses of relapsed SCLC samples and matched models subsequently identified treatment-emergent or enriched YAP1-associated non-neuroendocrine, epithelial-to-mesenchymal transition (EMT), and drug-tolerant persister-like states, accompanied by lower DLL3 and SEZ6 and relative preservation of B7-H3 and TROP2 (10). These observations support reassessment of tumor state at relapse rather than assuming persistence of the diagnostic subtype. Other multi-omic studies and reviews likewise emphasize neuroendocrine/non-neuroendocrine heterogeneity and suggest that treatment-associated plasticity may intersect with resistance, immune regulation, and target evolution (28–30).

### 4.2 MHC-I/APM and tumor immune visibility

Despite a high mutational burden, SCLC frequently displays limited antigen presentation. In a translational analysis of CheckMate 032, Rudin et al. found that higher antigen presentation machinery (APM)-related features and greater CD8 infiltration were associated with better outcomes following nivolumab-based therapy (26). Because this was an association within a treatment cohort, it does not by itself establish a predictive biomarker. More broadly, tumor antigenicity is not equivalent to immune visibility: the presence of potential tumor antigens does not guarantee effective presentation through MHC-I to cytotoxic T cells.

Mechanistic studies suggest that antigen-presentation capacity in SCLC is regulated at multiple levels. CRACD loss can suppress MHC-I through EZH2-related epigenetic mechanisms in preclinical models (31). LSD1 inhibition increases MHC-I/APM expression and enhances T-cell killing and checkpoint-blockade activity in experimental systems (32). Studies of ERBB2 signaling that integrate human samples and mouse models suggest a role in MHC-I suppression, with pathway intervention restoring antigen presentation in models (33). Additional preclinical work links ferroptosis-related programs to antigen-presentation regulation (34). Collectively, these data support viewing SCLC antigen presentation as a potentially dynamic and modifiable state, while the clinical tractability of these mechanisms remains to be established.

### 4.3 Microenvironment remodeling and its relationship to platinum resistance

The SCLC tumor microenvironment (TME) includes lymphoid and myeloid cells, fibroblasts, vascular cells, extracellular matrix, and soluble mediators. Their abundance and spatial organization vary between tumors and treatment states. SCLC profiling linked endothelial-to-mesenchymal transition and stromal programs to platinum resistance, illustrating that an inflammation-associated subtype can also contain adverse stromal features (28). Consequently, an immune-rich expression signature should not be equated with effective antitumor immunity.

Tumor-associated macrophages (TAMs) may provide trophic signals and suppress effector immunity, although their states extend beyond a simple M1/M2 classification. In SCLC cell and xenograft experiments, macrophage-derived IL-6 and cell contact promoted STAT3 activation and tumor growth (35). This is direct SCLC mechanistic evidence, but it does not establish that macrophage-directed treatment reverses clinical platinum resistance. Human bronchoalveolar-lavage analyses associated IL-10-producing M2-like macrophages with regulatory T cells (Tregs) and disease features (36); these observations are associative. Tregs and myeloid-derived suppressor cells (MDSCs) are relevant candidate constraints on T-cell competence, but their measurement is not a validated treatment-selection test in relapsed SCLC. In a clinical and translational study of PD-L1/TGF-β blockade in relapsed small-cell cancers, suppressive immune populations were associated with hyperprogression (37). The mixed population and specific treatment context limit generalization to platinum resistance.

Cytokines should likewise be interpreted by source and context. IL-6 can activate survival signaling; experimental NSCLC evidence connects this pathway to apoptosis and DNA-repair responses during cisplatin treatment, but that causal resistance mechanism is extrapolative for SCLC (38). IL-10-associated macrophage/Treg states provide an SCLC-specific example of immune suppression (36). TGF-β can inhibit effector immunity and participate in stromal remodeling, yet tumor-intrinsic TGF-β signaling can also restrain proliferation in a subset of SCLC. The hyperprogression study therefore argues against treating TGF-β as a uniformly detrimental pathway or recommending indiscriminate blockade (37).

Hypoxia, abnormal vasculature, and matrix remodeling may affect drug delivery and spatial immune exclusion. Direct SCLC evidence is strongest for associations between stromal or endothelial programs and resistance; the full causal sequence from hypoxia to platinum failure or ADC delivery failure remains incompletely resolved (28, 29). Platinum-containing treatment can reduce tumor burden and expose antigens while also selecting surviving tumor states and perturbing immune-cell composition. The balance between immune activation and suppression is not fixed, and cross-sectional specimens cannot establish that a particular microenvironment was caused by platinum (8, 10, 28). Paired pretreatment and relapse sampling is needed to distinguish treatment-induced changes from pre-existing heterogeneity.

These processes have different implications across modalities. Checkpoint blockade requires an effective tumor-reactive immune response; TCEs bypass classical peptide–MHC recognition but still require T-cell access and function. ADCs can deliver cytotoxic agents without relying on antigen-specific T-cell recognition, yet vascular access, target distribution, delivery, and payload susceptibility may remain limiting. Thus, an immune-suppressive TME does not itself prove ADC resistance, and an immune-inflamed TME does not guarantee ICI or TCE benefit. TME measures belong within the exploratory Immune dimension and, for drug access, the delivery context of the Target–Payload Pair (22, 26, 39, 40).

### 4.4 From immune visibility to functional immune state

Restoration of MHC-I alone is unlikely to explain the full range of responses to immunotherapy. Even when tumor antigens are presented, antitumor immunity may remain limited if T cells are not available for recruitment or are functionally impaired. Within the Time–Immune–Target framework, we therefore define Immune as a functional immune state encompassing tumor visibility, effector availability, effector competence, and immune contexture. This definition integrates transcriptional state, antigen presentation, and T-cell function; it is not intended to represent a validated standalone biomarker.

## 5 Why conventional immune checkpoint inhibition has limited activity in relapsed SCLC

Checkpoint blockade is most effective when an antitumor immune response already exists but is restrained by inhibitory signaling. Blocking PD-1/PD-L1 or CTLA-4 can release that restraint, yet it cannot reliably replace absent antigen presentation, generate tumor-infiltrating T cells *de novo*, or restore severely impaired effectors. These upstream limitations provide a biological explanation for why single-agent checkpoint inhibition may be inadequate in many relapsed SCLC tumors.

This mechanistic limitation is consistent with clinical experience. In CheckMate 331, nivolumab did not improve OS compared with chemotherapy in relapsed SCLC; median OS was approximately 7.5 versus 8.4 months, and PFS was shorter with nivolumab (41). These findings do not imply that SCLC is uniformly unresponsive to ICIs. In third-line nivolumab studies, ORR was approximately 11.9%, with some responses being durable (42), while pooled KEYNOTE-028/158 analyses reported an ORR of approximately 19.3% with pembrolizumab, again with durable benefit in a minority of patients (43).

These historical salvage ICI studies largely preceded routine first-line PD-L1 treatment and should not be used to infer the efficacy of ICI rechallenge after contemporary chemoimmunotherapy (41–43).

The more appropriate interpretation is therefore not that relapsed SCLC is categorically resistant to immunotherapy, but that unselected populations are not sufficiently enriched for immune states most likely to support effective checkpoint blockade. This limitation has motivated therapies that are less dependent on pre-existing tumor recognition and instead actively redirect T cells toward the tumor, including DLL3-directed T-cell engagers.

## 6 DLL3-directed T-cell engagement: rebuilding the tumor–T-cell cytotoxic connection

### 6.1 T-cell engagers alter the dependence on immune recognition

DLL3 is frequently detected in SCLC and is comparatively restricted on the surface of normal tissues. A DLL3×CD3 bispecific molecule connects DLL3-bearing tumor cells with CD3-positive T cells, thereby promoting immune-synapse formation and cytotoxic killing; studies of AMG 757, now known as tarlatamab, established this rationale preclinically (22). Unlike checkpoint inhibitors, this approach can reduce reliance on classical MHC-I-restricted tumor recognition, but it cannot dispense with either an accessible tumor target or competent T cells. Across systematic reviews and tissue series, the prevalence of DLL3 is high, whereas staining intensity, assay definitions, and spatial distribution vary (44, 45). Thus, DLL3 should be interpreted as a prevalent but nonuniform target whose availability may evolve, rather than as a fixed positive-or-negative selector (46).

### 6.2 From phase I to randomized phase III evidence

Clinical development of tarlatamab began with a phase I study in 107 previously treated patients with SCLC. The ORR was approximately 23.4%; median DOR, PFS, and OS were 12.3, 3.7, and 13.2 months, respectively, and CRS emerged as the signature treatment-related toxicity (47). At the recommended dose of 10 mg every 2 weeks, DeLLphi-301 subsequently reported an ORR of about 40% and a median PFS of about 4.9 months (48). An FDA analysis using a later cutoff evaluated 99 patients, again finding a 40% ORR and estimating median DOR at 9.7 months (49). Because the regulatory analysis used updated follow-up, its DOR estimate should not be conflated with that of the original publication.

Randomized evidence followed in DeLLphi-304, which assigned 509 patients to tarlatamab or the physician’s choice of topotecan, lurbinectedin, or amrubicin. Overall survival favored tarlatamab: median OS was 13.6 months compared with 8.3 months for control (hazard ratio, 0.60). Median PFS was 4.2 versus 3.2 months, and the corresponding ORRs were approximately 35% and 20%. Grade 3 or higher adverse events occurred less often overall with tarlatamab than with chemotherapy (5). These results move DLL3-directed T-cell engagement beyond single-arm activity and establish a phase III survival advantage in relapsed SCLC.

### 6.3 Target availability × T-cell fitness: beyond simple DLL3 positivity

The relevant question for T-cell engagement extends beyond whether DLL3 is present. In a small prospective study, Mishra et al. found that the proportion of DLL3-positive CTCs was associated with tarlatamab response; a threshold of ≥25% DLL3-positive CTCs showed promising discriminatory performance in that cohort, but the sample size was small and the threshold requires independent validation (50). Regarding acquired resistance, small translational studies have observed progression associated with reduced DLL3 in some cases, whereas other cases retained DLL3 but showed changes in systemic T-cell functional state (50, 51). We use these observations to distinguish target-side and immune-effector-side bottlenecks as a mechanistic framework rather than as validated resistance categories.

TCE activity can therefore be considered in terms of accessible target and functional effectors. In a peer-reviewed report involving two patients treated with tarlatamab plus an anti-PD-1 antibody, progression biopsies showed low DLL3 expression, phenotypic change, exhausted CD8-positive T cells, and enrichment of suppressive immune cells (52). The combination-treatment context, two-patient sample, and observational design prevent attribution to tarlatamab alone or validation of a resistance classifier. These findings support exploratory study of target and effector constraints without a subtype-specific treatment rule.

### 6.4 Safety, CNS disease, and combination strategies

CRS and immune effector cell-associated neurotoxicity syndrome (ICANS) create management requirements that distinguish T-cell engagers from conventional chemotherapy and ADCs. Available data indicate that most CRS events occur early during treatment, making step-up dosing, short-term intensified monitoring, and supportive care central to safe implementation of tarlatamab (53). An update from DeLLphi-300 observed intracranial antitumor activity in a subset of patients with brain metastases, although the analysis was not a dedicated randomized CNS study and requires confirmation in larger cohorts (54). DeLLphi-303 provided early safety and activity data for tarlatamab combined with PD-L1 blockade as first-line maintenance therapy. Although conducted outside the relapsed setting and non-randomized, the study provides early clinical support for the feasibility and mechanistic complementarity of T-cell redirection plus checkpoint blockade (55).

Within the Time–Immune–Target framework, T-cell engager activity is more directly conditioned by target availability and T-cell function than by prior platinum response alone. Platinum sensitivity therefore does not fully capture the biological requirements of DLL3-directed T-cell engagement.

## 7 Antibody–drug conjugates: from target expression to the Target–Payload Pair

T-cell engagers illustrate why treatment sensitivity cannot be inferred from relapse timing alone. Although surface antigens provide an entry point for both TCEs and ADCs, their downstream requirements differ. TCEs depend on target accessibility and T-cell function. Conventional ADC delivery involves antigen binding, internalization and trafficking, linker processing, and release of an active payload; delivery and cellular payload susceptibility must be considered together (39, 40).

### 7.1 ADC efficacy cannot be explained by target positivity alone

For conventional ADC delivery, the antibody must reach and bind an accessible antigen, after which internalization and intracellular processing release a payload into susceptible cells. This is an important pathway rather than an absolute requirement for every ADC in every setting. Experimental work with T-DXd outside SCLC also demonstrates effective extracellular payload release and interactions with the TME (39). The relevance of alternative release pathways to individual SCLC ADCs remains to be established. Target staining alone cannot summarize delivery efficiency or payload susceptibility (40).

B7-H3 is among the most intensively developed ADC targets in SCLC. Large molecular datasets suggest that B7-H3 is broadly expressed across SCLC molecular subtypes and may be less restricted by neuroendocrine lineage than DLL3 or SEZ6 (56). B7-H3 expression may also be associated with immune contexture; a retrospective study linked higher B7-H3 expression to impaired CD8 effector features and shorter PFS with chemo-immunotherapy, but these observations should not be interpreted as evidence that B7-H3 expression predicts ADC benefit (57).

### 7.2 I-DXd: clinical activity without a validated B7-H3 expression threshold

I-DXd links B7-H3 to the DXd TOP1-inhibitor payload. IDeate-Lung01 was a two-part, open-label phase II study: part 1 randomized patients to 8 or 12 mg/kg, and part 2 expanded 12 mg/kg. There was no standard-treatment control. In the pooled 12-mg/kg cohort (n=137), centrally assessed ORR was 48.2%, median DOR 5.3 months, PFS 4.9 months, and OS 10.3 months; OS follow-up in part 2 was still immature. The second-line subgroup (n=32) had ORR 56.3% and median PFS 5.6 months. Adjudicated treatment-related ILD occurred in 12.4%, including grade ≥3 events in 4.4% (6). Earlier phase I/II evidence also showed SCLC activity (58). These results describe activity and toxicity without supporting cross-trial efficacy ranking.

Regulatory status update beyond the core literature cutoff: on 25 September 2026, the sponsors voluntarily withdrew the US application seeking accelerated approval for I-DXd after FDA discussions indicated that the submitted evidence did not meet accelerated-approval requirements. Phase III evaluation continued (59). This administrative update does not negate the phase II activity estimates or establish clinical benefit relative to a control treatment.

Importantly, IDeate-Lung01 did not identify a clinically meaningful relationship between B7-H3 expression and outcome (6). The single-arm phase I/1b study of another B7-H3-directed ADC, YL201, likewise reported substantial response activity but did not establish a reliable expression threshold in the limited biomarker-evaluable population (60). The Target dimension should therefore not be reduced to a single baseline IHC-positive versus IHC-negative measurement; expression intensity, spatial heterogeneity, internalization efficiency, and payload biology may all influence therapeutic activity.

### 7.3 ADC resistance should be dissected into target-side and payload-side mechanisms

Evidence from ADC-treated cancers outside SCLC helps separate two broad routes to resistance. On the target side, DAISY associated intratumoral heterogeneity and treatment-related target change with outcome (61). Work by Chen et al. using paired clinical samples and experimental models additionally implicated lower target abundance, changes at antibody-binding sites, and defective internalization (62). Because these observations arose in other malignancies, they should inform SCLC-specific hypotheses rather than be presented as established SCLC resistance pathways.

Resistance can also reside downstream of target binding. Breast cancer models studied by Rampa et al. developed payload-dependent resistance features, and sensitivity was partly recovered when the payload mechanism was changed (63). In HER2-mutant NSCLC, resistance to the DXd payload was associated with reduced SLFN11 and drug export mediated by ABCC1/MRP1 (64). Neither study validates SLFN11, ABCC1/MRP1, or payload cross-resistance as a predictive marker for a TOP1-payload ADC in SCLC; their relevance here remains an extrapolation.

These findings suggest that the functional unit for interpreting an ADC is not the antigen alone but the combination of target and payload. Sequential products directed at different surface antigens may still share a resistance liability if they deliver the same TOP1/DXd payload class. Whether this proposed Target-Payload Pair meaningfully predicts sequencing outcomes in SCLC has not yet been tested directly. The Target–Payload Pair also encompasses delivery and is a simplifying concept: antibody properties, linker chemistry, drug-to-antibody ratio, distribution, dose, and toxicity can differ between products (40).

### 7.4 Potential immunologic effects of DXd-based platforms require validation in SCLC

Experimental studies conducted largely outside SCLC indicate that some DXd-based ADCs can trigger features of immunogenic cell death and alter the surrounding immune microenvironment (39, 40, 65, 66). Other preclinical combinations of ADCs with immune-engaging approaches provide additional support for possible immunologic cooperation (67). These data justify investigation, but they neither show that immune modulation accounts for the clinical activity of I-DXd in SCLC nor establish a currently appropriate combination regimen.

## 8 SEZ6 and DLL3 ADCs in the Target–Payload Pair

### 8.1 SEZ6 expression and lineage state

SEZ6 is a neuronal and neuroendocrine-associated membrane protein that is highly expressed in SCLC and associated with ASCL1- and NEUROD1-related neuroendocrine lineage states. Large clinical datasets show overall high but subtype-heterogeneous SEZ6 expression in SCLC (68), whereas studies of relapsed disease suggest that SEZ6 may decrease in treatment-emergent YAP1-associated states (10). These data support considering SEZ6 a lineage-associated therapeutic surface target rather than assuming that expression remains stable throughout the disease course. Real-world multi-omic analyses likewise identify associations between surface therapeutic targets and transcriptional state in SCLC (69).

### 8.2 SEZ6 ADCs with distinct payloads

ABBV-011 targets SEZ6 and delivers calicheamicin. Preclinical SCLC models demonstrated internalization and antitumor activity (70). In phase I, 99 patients received monotherapy and 98 were efficacy evaluable; ORR was 19% (19/98). In the 1-mg/kg expansion cohort (n=40), ORR was 25%, median DOR 4.2 months, and PFS 3.5 months. Hepatic abnormalities and two cases of veno-occlusive liver disease were reported (71).

ABBV-706 links SEZ6 to a TOP1 inhibitor. The open-label phase I program included dose escalation, randomized dose optimization, and expansion, without a standard-treatment control. Among 124 SCLC monotherapy patients, ORR was 52% (65/124), median DOR 5.3 months, PFS 5.4 months, and OS 11.3 months. The randomized 1.8-mg/kg cohort (n=41) had ORR 56% (23/41). A second-line subset (n=17) and SEZ6/ctDNA analyses were exploratory (7).

ABBV-011 and ABBV-706 therefore illustrate that the same target can be paired with different payloads and yield distinct observed clinical profiles. They do not, however, constitute a head-to-head comparison, and differences in patient populations, dose, development stage, and payload preclude attribution of efficacy or safety differences to the payload itself.

### 8.3 Prior TOP1 exposure and sequencing uncertainty

At 1.8 mg/kg, a *post hoc* analysis found ORR of 42% after conventional TOP1-inhibitor treatment versus 62% without such exposure. The protocol excluded prior TOP1-payload ADCs; these data cannot establish ADC-to-ADC cross-resistance (7). Confounding by treatment line and disease history remains possible. Cross-tumor payload-resistance studies provide mechanistic hypotheses rather than validated SCLC sequencing rules (63, 64).

### 8.4 DLL3 ADCs and the limits of target-only interpretation

Rovalpituzumab tesirine (Rova-T) coupled a DLL3-directed antibody to a pyrrolobenzodiazepine dimer payload. In the single-arm phase II TRINITY study of third-line or later DLL3-expressing SCLC (n=339), centrally assessed ORR was 12.4% and median OS was 5.6 months (72). In the randomized phase III TAHOE study of second-line DLL3-high SCLC (n=444), median OS was inferior with Rova-T compared with topotecan: 6.3 versus 8.6 months (hazard ratio, 1.46; 95% CI, 1.17–1.82). Serosal effusions, photosensitivity, and peripheral edema were more frequent with Rova-T (73). High target expression therefore did not ensure a favorable therapeutic index.

The contrasting development histories of DLL3-directed Rova-T and tarlatamab show that a target cannot be evaluated independently of the therapeutic platform (5, 73). A TCE requires a functional immune synapse; a DLL3 ADC must deliver an effective, tolerable payload. These studies are not a head-to-head comparison and cannot isolate payload effects, but they refute the assumption that failure of one DLL3-directed product invalidates every DLL3-directed modality. Similarly, ABBV-011 and ABBV-706 combine SEZ6 with distinct payload classes, whereas I-DXd combines B7-H3 with DXd. The biological requirements, potential resistance mechanisms, representative toxicities, and evidence limitations of ICIs, TCEs, and ADCs are summarized in **Table 2**. **Table 3** compares these target–payload combinations without ranking efficacy across trials.

**Table 2:** Mechanisms and evidence limits of ICI, TCE, and ADC treatment.

Modality	Requirements	Candidate measures	Failure bottlenecks	Representative toxicities	Evidence and interpretation
ICI	Antigen presentation and tumor-reactive effectors	MHC-I/APM, CD8 and SCLC-I; exploratory	Immune invisibility, exclusion or effector dysfunction	Immune-mediated pneumonitis, colitis and endocrinopathy	First-line atezolizumab/durvalumab combinations; historical salvage ICI evidence does not validate rechallenge (23, 24, 26, 41–43)
DLL3 TCE	Accessible DLL3 and functional T cells	DLL3 IHC/CTCs and T-cell state; exploratory	Target loss or reduced access; dysfunctional effectors	CRS and ICANS; step-up dosing and monitoring	Tarlatamab has randomized second-line OS evidence; resistance observations remain limited (5, 50–53)
B7-H3 ADC	Target access, effective delivery and payload sensitivity	B7-H3 IHC, ctDNA and prior TOP1 exposure; exploratory	Binding/delivery failure; target heterogeneity or payload resistance, largely cross-tumor hypotheses	I-DXd: ILD/pneumonitis, nausea and cytopenias	I-DXd lacks a standard-treatment control; no validated expression cutoff or ADC sequencing rule (6, 39, 40, 61–64)
SEZ6 ADC	SEZ6 lineage state and effective payload delivery	SEZ6 IHC and ctDNA; exploratory	Lineage-associated target change; delivery or payload failure remains incompletely defined	ABBV-011: hepatic injury/VOD; ABBV-706: cytopenias and fatigue	Distinct SEZ6 payload platforms; no head-to-head attribution to payload alone (7, 10, 68, 70, 71)
DLL3 ADC	Accessible DLL3, payload delivery and therapeutic index	DLL3 selection did not ensure benefit	Target positivity cannot compensate for inadequate therapeutic index	Rova-T: serosal effusions, photosensitivity and edema	Rova-T was inferior to topotecan in TAHOE; failure does not invalidate DLL3 TCEs (72, 73)

This comparison organizes mechanisms, biological requirements, failure bottlenecks, representative toxicities, and evidence maturity. Candidate measurements are not established treatment-selection tests. Toxicities are product dependent and are not exhaustive.

**Table 3:** Selected ADC platforms in previously treated SCLC.

Agent	Target and payload	Study population	Activity estimates	Safety considerations	Framework interpretation
Rova-T	DLL3; PBD dimer	TRINITY: third-line or later, n=339; TAHOE: randomized second line, n=444	TRINITY: ORR 12.4%, OS 5.6 mo. TAHOE: OS 6.3 vs 8.6 mo; HR 1.46	Serosal effusions; photosensitivity; edema	Target-high selection did not ensure therapeutic index; not equivalent to DLL3 TCE activity (72, 73)
ABBV-011	SEZ6; calicheamicin	Phase I: 99 treated, 98 efficacy evaluable; 1-mg/kg expansion n=40	Overall ORR 19% (19/98). Expansion: ORR 25%, DOR 4.2 mo, PFS 3.5 mo	Hepatic abnormalities; two VOD cases	SEZ6 provides delivery context; payload and dose must be considered (70, 71)
ABBV-706	SEZ6; TOP1 inhibitor	Phase I: SCLC monotherapy n=124; randomized dose optimization	ORR 52%; DOR 5.3 mo, PFS 5.4 mo, OS 11.3 mo	Anemia and other cytopenias; fatigue	Prior conventional TOP1 exposure is exploratory; prior TOP1-payload ADCs excluded (7)
I-DXd	B7-H3; DXd TOP1 inhibitor	Phase II dose optimization then expansion; pooled 12 mg/kg n=137; second line n=32	Pooled: ORR 48.2%, DOR 5.3 mo, PFS 4.9 mo, OS 10.3 mo	ILD/pneumonitis; nausea; cytopenias	No validated B7-H3 expression threshold; delivery and payload matter (6)

Figures refer to different trial designs, doses, prior treatments, and assessment methods; no cross-trial ranking is intended. Time describes relapse and exposure history; Immune captures contextual interactions; Target–Payload includes effective delivery and payload susceptibility. None of these ADC targets is a validated universal marker of platinum or ICI resistance. Regulatory context for I-DXd is discussed separately in Section 7.2.

For each ADC, Time records relapse kinetics and prior treatment, Immune describes contextual immune interactions rather than an obligatory T-cell recognition step, and Target captures accessible antigen together with the delivery pathway. Payload susceptibility is nested within the Target–Payload Pair, not inferred from antigen positivity. The pair is shorthand: antibody properties, linker stability, drug-to-antibody ratio, tissue distribution, and release kinetics also contribute to efficacy and toxicity. Neither DLL3, B7-H3, nor SEZ6 should be described as a validated marker of platinum or ICI resistance that automatically selects an ADC (6, 7, 71, 73).

## 9 Time–immune–target: a multidimensional framework for relapsed SCLC

Current evidence supports retaining platinum sensitivity while redefining its scope. Time most directly captures relapse kinetics, prior platinum response, and the plausibility of platinum rechallenge. It does not independently determine whether the relapsed tumor retains an actionable target, presents an exploitable immune state, or has developed a mechanism-specific form of resistance.

The Immune dimension focuses on functional immune state. For ICIs, relevant conditions include MHC-I/APM, T-cell infiltration, and pre-existing antitumor immunity; for T-cell engagers, classical MHC-I dependence can be partly bypassed, but functional T cells remain necessary. The Target dimension emphasizes therapeutically accessible targets at relapse rather than relying solely on expression in historical specimens. For ADCs, target properties must additionally be interpreted together with payload biology and the delivery process.

This reframes resistance around the question: Resistance to what mechanism? Platinum resistance primarily describes the clinical relapse phenotype after prior platinum therapy. Reduced DLL3 may constitute a target-side constraint on T-cell engagement, whereas impaired T-cell function may represent an immune-effector constraint. For ADCs, loss of SLFN11 and drug efflux have been proposed as candidate payload-side resistance mechanisms in cross-tumor studies but have not been directly validated in SCLC (63, 64). These mechanisms may overlap, but none should be collapsed into a single, universal label of treatment resistance.

Treatment history is not a fourth independent dimension. Rather, it is the clinical and evolutionary context that overlays Time, Immune, and Target. Different therapies may impose distinct selective pressures on subsequent tumor states, but the magnitude and predictive relevance of these effects must be validated separately for each treatment class rather than inferred from exposure alone.

Time-Immune-Target is proposed as an organizing model, not as a requirement for universal testing or a prescriptive treatment pathway. Its function is to identify which constraint may be most relevant for a given modality. A long PFI and a strong previous platinum response may favor reconsideration of platinum; DLL3 accessibility and T-cell competence define different prerequisites for a T-cell engager; and an ADC additionally relies on effective payload delivery and payload susceptibility, commonly through internalization and intracellular processing. Prior conventional TOP1-inhibitor treatment and prior exposure to a TOP1-payload ADC should be recorded separately, because neither variable has prospective treatment-selection validation in SCLC. These examples illustrate how the model can structure research questions without creating clinical rules.

The intended advance is therefore not another immutable SCLC taxonomy. Instead, the framework links each modality to the biological conditions needed for it to work, an approach we term precision modality selection. **Figure 2** contrasts these requirements across the principal therapeutic classes considered here.

**Figure 2:**
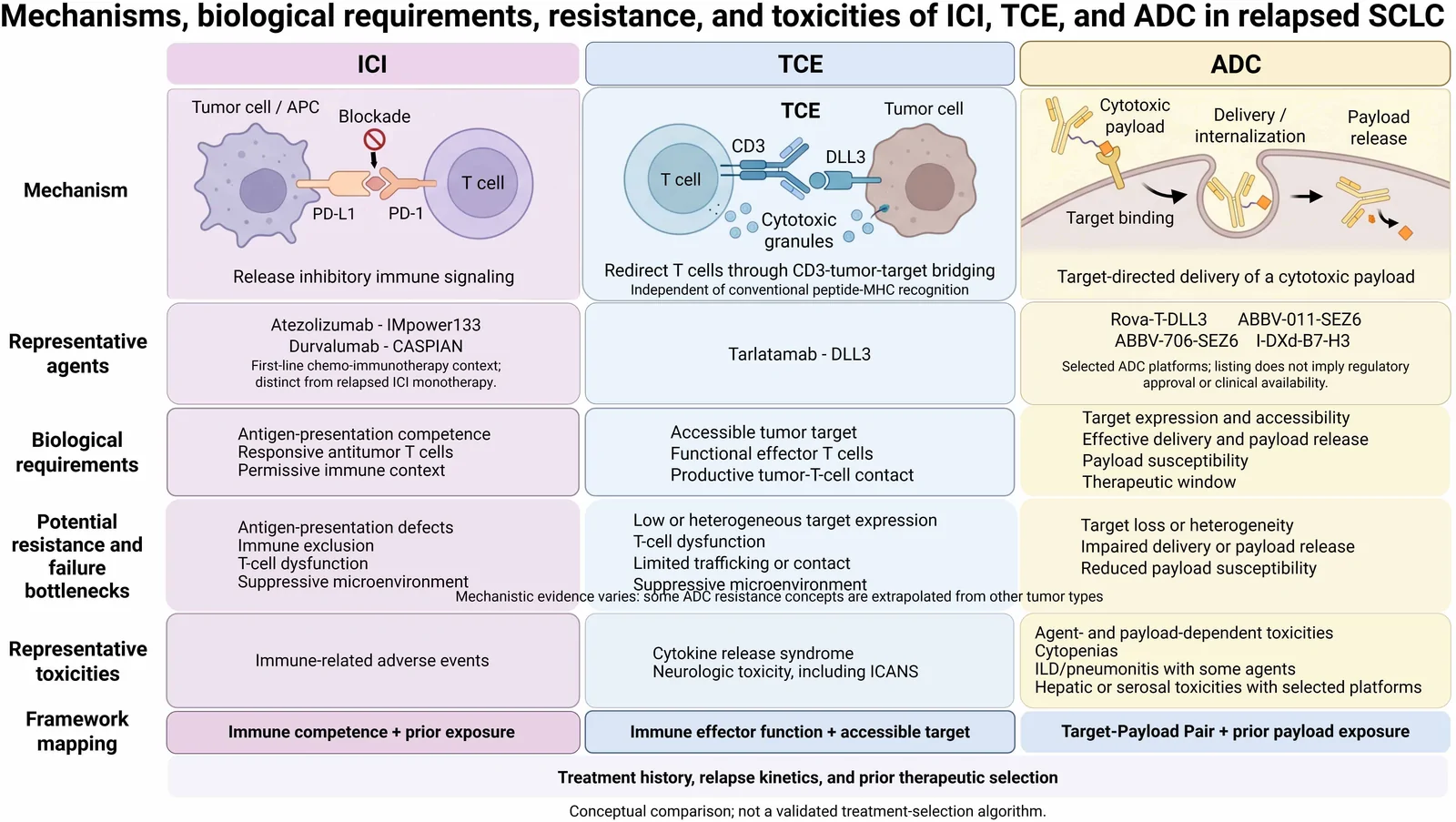
Mechanisms, biological requirements, resistance, and toxicities of ICI, TCE, and ADC in relapsed SCLC. The three therapeutic modalities are compared by mechanism, representative agents, biological requirements, potential resistance and failure bottlenecks, representative toxicities, and framework mapping. ICIs block inhibitory immune signaling and depend on antigen-presentation competence and responsive antitumor T cells. Atezolizumab (IMpower133) and durvalumab (CASPIAN) are shown as first-line chemo-immunotherapy context, distinct from relapsed ICI monotherapy. Tarlatamab illustrates DLL3-directed T-cell engagement through CD3–tumor-target bridging without conventional peptide–MHC recognition; accessible targets, functional effector T cells, and productive tumor–T-cell contact remain relevant. ADCs illustrate target binding, delivery/internalization, and release of the cytotoxic payload, not release of an intact therapeutic antibody; internalization represents a common delivery route. Rovalpituzumab tesirine (Rova-T; DLL3), ABBV-011 and ABBV-706 (SEZ6), and ifinatamab deruxtecan (I-DXd; B7-H3) are selected platforms, and their listing does not imply regulatory approval or clinical availability. Rova-T is a historical negative clinical example. ADC resistance evidence varies, with some concepts extrapolated from other tumor types; toxicities are agent- and payload-dependent. Prior treatment and relapse kinetics provide context across all modalities. This is a conceptual comparison, not a validated treatment-selection algorithm. APC, antigen-presenting cell; ICANS, immune effector cell-associated neurotoxicity syndrome; ILD, interstitial lung disease. Created in BioRender. YANG, S. (2026) https://BioRender.com/5i1bxcu.

## 10 Discussion and future directions: toward a testable dynamic treatment model

### 10.1 From single baseline specimens to longitudinal, multimodal state monitoring

Future biomarker research should move from single baseline measurements toward longitudinal monitoring. Tissue biopsy can characterize transcriptional state, MHC-I/APM, and target expression but is limited by sampling location. CTCs and ctDNA may provide dynamic information, while molecular imaging may help capture whole-body spatial heterogeneity. In the first-in-human DLL3 immunoPET study by Tendler et al., DLL3-avid lesions were identified in 12 of 15 patients in the expansion cohort, with marked inter-patient, intra-patient, and inter-site heterogeneity in uptake (74). These data provide early proof of concept for whole-body target imaging but do not establish that DLL3 immunoPET can prospectively select patients for tarlatamab or improve treatment sequencing. Beyond conventional tissue and liquid biomarkers, computational pathology may offer a scalable approach to quantifying the tumor microenvironment. In lung adenocarcinoma, Zhang et al. used HoVer-Net-based segmentation of routine pathological images to quantify epithelial cells, lymphocytes, macrophages, and neutrophils and linked cellular composition to nodal status and prognostic stratification (75). Although this evidence is cross-tumor and does not establish an SCLC biomarker, it illustrates how digital pathomics could complement longitudinal tissue, blood, and imaging measurements in future multidimensional SCLC studies.

### 10.2 From prognostic association to predictive validation

SCLC has many candidate biomarkers associated with outcome, but association with outcome alone does not establish treatment-predictive value. Iasonos et al. emphasized that a predictive biomarker requires evidence of differential relative treatment benefit across biomarker-defined groups, that is, a biomarker–treatment interaction (76). On this basis, SCLC-I, APM/CD8, DLL3-positive CTCs, B7-H3/SEZ6 expression, and ctDNA dynamics should currently be regarded primarily as associated, exploratory, or candidate biomarkers rather than validated predictive biomarkers. Prior conventional TOP1-inhibitor exposure is more accurately categorized as an exploratory treatment-history variable, while the predictive relevance of prior TOP1-payload ADC exposure in SCLC lacks direct clinical evidence.

### 10.3 Combination strategies should address defined biological bottlenecks

Combination hypotheses should, where possible, arise from explicit mechanistic complementarity. If DLL3 remains present after T-cell engager therapy but T-cell effector function becomes limiting, combining T-cell redirection with immune modulation has a mechanistic rationale and early clinical feasibility support (55). If ADC resistance in a given context is driven by payload efflux or altered DNA-damage response, cross-tumor models suggest that payload diversification may be more mechanistically rational than simply switching the surface target while retaining the same payload (63, 64). These concepts should not be extrapolated directly into standard SCLC sequencing strategies. Future trials should prospectively define the biological bottleneck they intend to address and incorporate corresponding translational endpoints. Microenvironment-directed combinations may also extend beyond checkpoint-based immune modulation. In a prospective, single-arm, multicenter phase II study of treatment-naive SCLC, Zhao et al. evaluated a vascular-normalization-oriented schedule of endostatin plus platinum–etoposide; among 21 evaluable patients, the ORR was 61.9%, median PFS was 8.0 months, and median OS was 13.6 months (77). Because the study had no comparator, included a small first-line population, and did not directly measure immune remodeling, it cannot establish treatment benefit or inform relapsed-SCLC sequencing; nevertheless, it provides clinical precedent for investigating angiogenic and vascular components of the tumor microenvironment as potential combination targets.

### 10.4 Sequencing research should record the history of mechanism-specific resistance

Treatment-sequencing studies should distinguish prior conventional TOP1-inhibitor chemotherapy from prior TOP1-payload ADC exposure. In IDeate-Lung01, ORR was 17/44 (38.6%) after previous TOP1-inhibitor treatment and 49/93 (52.7%) without it, but the investigators reported no clinically meaningful subgroup difference (6). The ABBV-706 *post hoc* association was likewise non-randomized and excluded prior TOP1-payload ADC exposure (7). Neither finding establishes a treatment-predictive effect or clinically meaningful ADC-to-ADC cross-resistance in SCLC. Future studies should prespecify these distinct exposure variables and incorporate progression sampling (63, 64).

### 10.5 Prospective validation of time–immune–target

Time–Immune–Target ultimately requires prospective studies capable of testing interactions rather than retrospective correlations. Ideally, such studies would record continuous PFI/CTFI and specific prior treatment classes, collect tissue, blood, and imaging biomarkers, and test whether these variables modify the relative benefit of different treatment modalities. A dimension becomes relevant for treatment selection only when it predicts differential benefit of treatment A relative to treatment B.

## 11 Limitations

This narrative review did not use a preregistered systematic search, formal risk-of-bias tool, or meta-analysis; literature selection and interpretation may therefore be affected by selection bias. Trials differ in treatment line, relapse definitions, prior ICI or cytotoxic exposure, target assays, dose, efficacy assessment, and follow-up. Response and survival estimates from unrelated studies should not be used to rank therapies, and results in fit trial participants may not extend to patients with substantial comorbidity or uncontrolled CNS disease.

Much of the biomarker literature is exploratory or associative. Associations between outcome and subtype, target expression, immune features, or ctDNA dynamics do not by themselves demonstrate differential relative benefit between treatments. Small translational cohorts and limited paired sampling further constrain inference about acquired resistance. Several ADC delivery and payload-resistance mechanisms discussed here come from breast cancer or NSCLC and remain hypotheses in SCLC; evidence for overlap with platinum resistance is incomplete.

Time–Immune–Target is an unvalidated conceptual framework. Its dimensions overlap, have no established weights or universal assay thresholds, and may vary across lesions and time. The Target–Payload Pair also simplifies a more complex delivery system. No prospective study has shown that assigning therapy using this framework improves outcomes. Its intended role is to organize research, prioritize longitudinal measurements, and motivate biomarker-by-treatment interaction analyses, rather than to direct routine clinical care.

## 12 Conclusion

Platinum sensitivity remains useful in relapsed SCLC, especially when considering platinum rechallenge, but relapse timing cannot summarize every subsequent therapeutic vulnerability. Five interconnected biological features provide a more complete account: lineage plasticity, antigen-presentation competence, T-cell function, surface-target availability, and payload susceptibility. Lineage transitions can reshape both immune visibility and lineage-associated surface antigens, linking the Immune and Target dimensions. Antigen presentation and T-cell function jointly influence checkpoint blockade, whereas TCEs can bypass classical MHC-I-restricted recognition but still require an accessible target and competent effectors. For ADCs, target availability must be considered with delivery and susceptibility to the released payload; this relationship is captured by the Target–Payload Pair.

Time–Immune–Target integrates these features with relapse kinetics and the selective pressures of prior therapy. It distinguishes resistance to platinum from resistance to immune recognition, T-cell redirection, or a particular ADC delivery–payload mechanism, while allowing that mechanisms may overlap. Serial tissue and blood sampling, spatial assessment, and prospective tests of biomarker–treatment interaction are required before this organizing framework can inform treatment assignment. Until then, it offers a basis for testable hypotheses rather than a validated clinical algorithm.
